# Effect of Ergodic and Non-Ergodic Fluctuations on a Charge Diffusing in a Stochastic Magnetic Field

**DOI:** 10.3390/e23060781

**Published:** 2021-06-19

**Authors:** Gerardo Aquino, Kristopher J. Chandía, Mauro Bologna

**Affiliations:** 1 Department of Computing, Goldsmiths, University of London, London WC1E 7HU, UK; aquigerardo@gmail.com; 2 Departamento de Ingeniería Eléctrica-Electrónica, Universidad de Tarapacá, Arica 1000000, Chile; kchandia@gmail.com

**Keywords:** fluctuating magnetic field, non-possonian processes, non-ergodic fluctuations

## Abstract

In this paper, we study the basic problem of a charged particle in a stochastic magnetic field. We consider dichotomous fluctuations of the magnetic field where the sojourn time in one of the two states are distributed according to a given waiting-time distribution either with Poisson or non-Poisson statistics, including as well the case of distributions with diverging mean time between changes of the field, corresponding to an ergodicity breaking condition. We provide analytical and numerical results for all cases evaluating the average and the second moment of the position and velocity of the particle. We show that the field fluctuations induce diffusion of the charge with either normal or anomalous properties, depending on the statistics of the fluctuations, with distinct regimes from those observed, e.g., in standard Continuous-Time Random Walk models.

## 1. Introduction

Diffusive processes occur in many physical, chemical and engineering applications. When the diffusion process is taking place, the quantities related to the spreading species take random values. Since Einstein and Smoluchowski’s work on Brownian motion [1,2], diffusive phenomena have been a fundamental subject of intense research. Both derivations (Einstein and Smoluchowski’s) lead to the well-known diffusion relationship, in the one-dimensional case, $\langle x^{2}\rangle =2Dt$, with *D* the diffusion coefficient. Several relevant physical and biological phenomena have been discovered in the last few decades, showing an anomalous relationship between mean-squared displacement and time, $\langle x^{2}\rangle \propto t^{\alpha }$. For example, diffusion through porous media or within a crowded cellular environment, making anomalous diffusion a relevant subject of research work [3,4,5,6] and Refs. [7,8] for a review.

This paper presents a detailed study of a particle moving in a fluctuating magnetic field that is directly connected to plasma physics. In particular, it is relevant in many technological applications such as, for example, plasma confinement [9]. We focus on the diffusion of the particles caused by the magnetic field fluctuations, which can destroy the plasma confinement. The fluctuations of physical quantities are an almost inevitable occurrence and generate diffusion processes of the related quantity [10,11,12]. It is important, therefore, to have an adequate theoretical framework to model their effect. We aim to fill the gap relative to the case of diffusion in a fluctuating magnetic field, which, to our knowledge, has not yet been explored and modeled so far in the case of non-ordinary statistics.

The paper is organized as follows: In Section 2 we introduce and formally define the problem for generic fluctuations. In Section 3, we introduce the case of dichotomous fluctuations, and we afford a complete analytical and numerical treatment of Poisson and non-Poisson statistics. In Section 4, we look at the average squared displacement and, starting from the Poisson case, we move to non-Poisson, power-law distributed fluctuations exploring, therefore also the non-ergodic regime when the average time of the fluctuations diverges (see Ref. [13] for an extended discussion, and Ref. [14] and references therein for a review and a historical perspective). The diffusion properties in this regime are characterized by the analytical and numerical derivation of the average squared displacement. Section 5 and Section 6 summarize results and draw final conclusions.

## 2. Stochastic Equation for the Magnetic Force

Let us consider the following classical equation [15,16]

(1) $$\begin{array}{c} m\frac{dv}{dt}=q\left(v\times B+E\right) \end{array}$$

where *m* is the mass of the charge *q*, v is the velocity, B the magnetic field, and E the electric field. We focus on the case where the particle is traveling in a region with a uniform magnetic field randomly fluctuating, i.e., $B=B_{0}+B_{1}\left(t\right)$ where $B_{1}\left(t\right)=B_{1}\xi \left(t\right)$ with ξ(t) the stochastic fluctuation of the magnetic field, where $\xi \left(t\right)=\xi \left(t\right)n_{\xi }$. We shall consider a dichotomous fluctuation, with values of ±1 where the sojourn time in one of the two states are distributed according to the distribution ψ(t). We will consider the case when ψ(t) is an exponential, ψ(t)=γexp[−γt], or Poisson case, and the case when ψ(t) is a power law, $\psi \left(t\right)\propto t^{-\alpha -1}$ with 0<α<2, or non-Poisson case.

The main reason for choosing dichotomous fluctuations rests on the fact that taking the finite values fluctuations may better represent a physical system. Additionally, we stress that in the appropriate limit, the most well-known noises in the literature, such as the gaussian white noise and the white shot noise are recovered within this framework [17,18]. Furthermore, we consider the case where no external electric field is applied. Taking the magnetic field in the *z* direction, $B\equiv B\left(t\right)k=[B_{0}+B_{1}\xi \left(t\right)]k$, and using cartesian coordinates, we have

(2) $$\begin{array}{ccc} & & \frac{dv_{x}}{dt}=\omega \left(t\right)v_{y}+\frac{q}{m}E_{x} \end{array}$$

(3) $$\begin{array}{ccc} & & \frac{dv_{y}}{dt}=-\omega \left(t\right)v_{x}+\frac{q}{m}E_{y} \end{array}$$

with ω(t)=qB(t)/m the time-dependent Larmor frequency and $E_{x}$, $E_{y}$ the induced electrical field. As a further simplification, we consider the time scale of the fluctuation much larger than the time associated with the unperturbed Larmor frequency $\omega_{0}=qB_{0}/m$. It is worthy to note that Equations (2) and (3) can be reduced to a second order stochastic differential equation for the function z=x+iy. The resulting equation has a strong similarity with the mechanical system studied in Refs. [19,20] where the authors study a linear damped oscillator with a noise perturbing both the oscillator mass and the friction. A detailed study of this equation is out of the scope of this paper, and it is left to an upcoming publication.

Neglecting the induced electrical field, E≈0, (see also [21]), we have

(4) $$\begin{array}{ccc} & & \frac{dv_{x}}{dt}=\omega \left(t\right)v_{y} \end{array}$$

(5) $$\begin{array}{ccc} & & \frac{dv_{y}}{dt}=-\omega \left(t\right)v_{x} \end{array}$$

For the sake of completeness, we end this section showing the equation for the density probability $P\left(v_{x}.v_{y},t\right)=P\left(v,t\right)$ associated with the stochastic Equations (4) and (5). To obtain a closed equation for P(v,t) we will assume that ξ is a Poisson process although, in the next sections, the analysis of the relevant quantiles, $\langle v_{x}\left(t\right)\rangle$, $\langle v_{y}\left(t\right)\rangle$, $\langle x{\left(t\right)}^{2}+y{\left(t\right)}^{2}\rangle$, will include also non-Poisson processes. Using the Liouville approach we write the following continuity equation

(6) $$\begin{array}{c} \frac{\partial \rho }{\partial t}=-\nabla \cdot \left[\rho v\times \omega \right]=-\nabla \cdot \left[\rho v\times \omega_{0}\right]-\nabla \cdot \left[\xi \rho v\times \omega_{1}\right] \end{array}$$

where for the sake of compactness we dropped the function arguments and we introduced the symbols

$$\nabla \equiv \frac{\partial }{\partial v_{x}}i+\frac{\partial }{\partial v_{y}}j,\;\;\omega =\left[\frac{qB_{0}}{m}+\frac{qB_{1}}{m}\xi \left(t\right)\right]k\equiv \omega_{0}+\omega_{1}\xi \left(t\right).$$

The stochastic density ρ is related to $P(v_{x}.v_{y},t)$ via the Van Kampen’s lemma [22] 〈ρ〉=P(v,t) where the average is performed on the ξ realizations. Additionally, we will use the Shapiro–Loginov formulae of differentiation [23]

(7) $$\frac{\partial }{\partial t}\langle \xi \left(t\right)\rho \left(t\right)\rangle =-\gamma \langle \xi \left(t\right)\rho \left(t\right)\rangle +\langle \xi \left(t\right)\frac{\partial }{\partial t}\rho \left(t\right)\rangle ,$$

that holds true for processes with a *n*th correlation function fulfilling the condition

$$\frac{\partial }{\partial t}\langle \xi \left(t\right)\xi \left(t_{1}\right)\cdots \xi \left(t_{n}\right)\rangle =-\gamma \langle \xi \left(t\right)\xi \left(t_{1}\right)\cdots \xi \left(t_{n}\right)\rangle .$$

In particular, this applies to Poisson, Gaussian and Markov jump processes, with a correlation function given by $\langle \xi \left(t_{1}\right)\xi \left(t_{2}\right)\rangle ∼\exp[-\gamma |t_{2}-t_{1}\left|\right]$. Taking the average of Equation (6), defining $P_{1}\left(v,t\right)\equiv \langle \xi \rho \rangle$ and using Equation (7), we may write the system

(8) $$\begin{array}{ccc} & & \frac{\partial P(v,t)}{\partial t}=-v\times \omega_{0}\cdot \nabla P-v\times \omega_{1}\cdot \nabla P_{1}, \end{array}$$

(9) $$\begin{array}{ccc} & & \frac{\partial P_{1}\left(v,t\right)}{\partial t}=-\gamma P_{1}\times \omega_{0}\cdot \nabla P_{1}+v\times \omega_{1}\cdot \nabla P \end{array}$$

Taking the time derivative of Equation (8), and after some algebra we obtain the following equation for the probability density

(10) $$\begin{array}{ccc} \frac{\partial^{2}P\left(v,t\right)}{\partial t^{2}} & = & -\gamma \frac{\partial P}{\partial t}-\gamma v\times \omega_{0}\cdot \nabla P+v\times \omega_{0}\cdot \nabla \left[v\times \omega_{0}\cdot \nabla P\right]+\\ & & -v\times \omega_{1}\cdot \nabla \left[v\times \omega_{1}\cdot \nabla P\right]. \end{array}$$

## 3. Dichotomous Processes

As stated in Section 2, in this section we will consider a magnetic field with a fluctuating component which is assumed to be dichotomous. Dichotomous fluctuations have the nice property of assuming finite values but, despite their relative simplicity, they can be shown to allow one to recover both gaussian white noise and white shot noise [17] within an appropriate limit procedure. Formally the exact solution of Equations (4) and (5) is

(11) vx(t)=v0sin∫0tω(u)du=v0Imexpiω0texpiω1∫0tξ(u)du,

(12) vy(t)=v0cos∫0tω(u)du=v0Reexpiω0texpiω1∫0tξ(u)du,

where $v_{0}$ is the initial velocity along the *y* axis. For the average we have

(13) 〈vx(t)〉=v0Imexpiω0texpiω1∫0tξ(u)du,

(14) 〈vy(t)〉=v0Reexpiω0texpiω1∫0tξ(u)du.

As we infer from Equations (13) and (14), we need to evaluate the average of the exponential of the noise integral. For this purpose, we consider the stochastic equation

(15) $$\frac{dU}{dt}=\xi \left(t\right)$$

with ξ(t) a dichotomous fluctuation where the sojourn time in one of the two states are distributed according to the distribution ψ(t). We assume that the event, occurring at each random time $t_{i}$, changes the ξ(t) sign. When these events occur with a constant rate γ this corresponds to a Poisson process, and is characterized by an exponential distribution. As stated in the Introduction, we will consider here both the case of exponential (Poisson) distribution with

(16) ψ(t)=γexp[−γt]

and the case of non-Poisson process with power-law distribution, characterized by the following asymptotic behavior

(17) $$\psi \left(t\right)\propto {\left(\frac{t}{T}\right)}^{-\alpha -1},\;\;\;\;t\gg T$$

with 0<α<2, which corresponds to a regime with a diverging second moment (1<α<2) and diverging first and second moment (0<α<1). The latter case, characterized by the absence of a finite time scale, corresponds to a condition of ergodicity breaking. The formal solution of Equation (15) is

(18) $$U\left(t\right)=\int_{0}^{t}\xi \left(u\right)du.$$

Considering the formal solution for the velocity of the charge, Equations (13) and (14), we need to evaluate the quantity $\langle \exp\left[i\omega_{1}\int_{0}^{t}\xi \left(u\right)du\right]\rangle$. For this purpose, we use the exact formula in the Laplace representation [24,25] (see Ref. [26] for detailed calculations)

(19) Lexpiω1∫0tξ(u)du=121+ψ^s−iω1Ψ^s+iω11−ψ^s−iω1ψ^s+iω1+1+ψ^s+iω1Ψ^s−iω11−ψ^s−iω1ψ^s+iω1=Re1+ψ^s+iω1Ψ^s−iω11−ψ^s−iω1ψ^s+iω1

where $\hat{\psi }\left(s\right)$ is the Laplace transform of ψ(t) and $\Psi \left(t\right)$ (and consequently $\hat{\Psi }\left(s\right)$ is its Laplace transform) is the probability that no switch occurs for a generic interval of time *t*, i.e.,

(20) $$\Psi \left(t\right)=1-\int_{0}^{t}\psi \left(t_{1}\right)dt_{1}=\int_{t}^{\infty }\psi \left(t_{1}\right)dt_{1}$$

The Poisson case does not present difficulties and, in the time representation, gives the expression

(21) $$\begin{array}{ccc} & & \langle \exp\left[i\omega_{1}\int_{0}^{t}\;\;\xi \left(u\right)du\right]\rangle =\\ & & \exp\left[-\frac{\gamma }{2}t\right]\left[\frac{\gamma \sinh\left(t\sqrt{\frac{\gamma^{2}}{4}-\omega_{1}^{2}}\right)}{2\sqrt{\frac{\gamma^{2}}{4}-\omega_{1}^{2}}}+\cosh\left(t\sqrt{\frac{\gamma^{2}}{4}-\omega_{1}^{2}}\right)\right]. \end{array}$$

We are now in position to write a closed expression for Equations (13) and (14). The average of the velocity components is

(22) $$\begin{array}{ccc} & & \langle v_{x}\left(t\right)\rangle =v_{0}\sin\left[\omega_{0}t\right]\\ & & \exp\left[-\frac{\gamma }{2}t\right]\left[\frac{\gamma \sinh\left(t\sqrt{\frac{\gamma^{2}}{4}-\omega_{1}^{2}}\right)}{2\sqrt{\frac{\gamma^{2}}{4}-\omega_{1}^{2}}}+\cosh\left(t\sqrt{\frac{\gamma^{2}}{4}-\omega_{1}^{2}}\right)\right]\\ & & \langle v_{y}\left(t\right)\rangle =v_{0}\cos\left[\omega_{0}t\right] \end{array}$$

(23) $$\begin{array}{ccc} & & \exp\left[-\frac{\gamma }{2}t\right]\left[\frac{\gamma \sinh\left(t\sqrt{\frac{\gamma^{2}}{4}-\omega_{1}^{2}}\right)}{2\sqrt{\frac{\gamma^{2}}{4}-\omega_{1}^{2}}}+\cosh\left(t\sqrt{\frac{\gamma^{2}}{4}-\omega_{1}^{2}}\right)\right] \end{array}$$

We now study Equation (19) for the non-Poisson case with a power-law distribution of the type of Equation (16) with 0<α<2 where the non-ergodicity of the process plays an important role. Some difficulties arise in inverting the Laplace transform, in particular in the region defined by 1<α<2. This is partially due to the fact that while for 0<α<1 the calculation in the Laplace transform can be carried out using as waiting-time distribution the derivative of the Mittag–Leffler function (see for example Ref. [8] and references therein), for the region 0<α<2 the Laplace transform is usually a complicated function, and the inversion of the final result is not an easy task. Traditionally the inversion of a Laplace transform for a large value of time t is performed using the Tauberian theorem, i.e., taking the development for the Laplace parameter s→0. If the function to invert is a hard-to-handle function, it is not always clear where to stop the development (see [27] for detailed examples). To overcome this difficulty, we may use as waiting-time distribution [28]

(24) $$\psi \left(t\right)=\frac{\sin\left(\frac{\pi \alpha }{2}\right)\cos\left(\frac{t}{T}\right)+\cos\left(\frac{\pi \alpha }{2}\right)\sin\left(\frac{t}{T}\right)}{T\cos\left(\frac{\pi \alpha }{2}\right)}-\frac{\sin\left(\frac{\pi \alpha }{2}\right)\cos_{\alpha }\left(\frac{t}{T}\right)+\cos\left(\frac{\pi \alpha }{2}\right)\sin_{\alpha }\left(\frac{t}{T}\right)}{T\cos\left(\frac{\pi \alpha }{2}\right)}.$$

where *T* is a time-scale parameter and, by definition [29,30],

(25) $$\begin{array}{c} \cos_{\alpha }t\equiv \frac{E_{\alpha }^{it}+E_{\alpha }^{-it}}{2},\;\;\sin_{\alpha }t\equiv \frac{E_{\alpha }^{it}-E_{\alpha }^{-it}}{2i} \end{array}$$

and

(26) $$E_{\alpha }^{t}\equiv D_{t}^{\alpha }\exp\left[t\right]=\sum_{n=0}^{\infty }\frac{t^{n-\alpha }}{\Gamma (n+1-\alpha )},$$

where $D_{t}^{\alpha }$ is the Riemann-Liouville fractional derivative. The functions $\cos_{\alpha }t$ and $\sin_{\alpha }t$ compensate the oscillatory behavior of the ordinary trigonometric functions, and what remains is a positive power law, i.e., $t^{-\alpha -1}$. A rigorous proof is given in Ref. [28]. Despite its complicated structure in time representation, its Laplace transform is

(27) $$\hat{\psi }\left(s\right)=\frac{1+sT\tan\left(\frac{\pi \alpha }{2}\right)-\sec\left(\frac{\pi \alpha }{2}\right){\left(sT\right)}^{\alpha }}{{\left(sT\right)}^{2}+1},\;\;0<\alpha <2,\;\;\alpha \neq 1.$$

For α=1 we must take the limit and we obtain

(28) $$\hat{\psi }\left(s\right)=\lim_{\alpha \to 1}\frac{1+sT\tan\left(\frac{\pi \alpha }{2}\right)-\sec\left(\frac{\pi \alpha }{2}\right){\left(sT\right)}^{\alpha }}{{\left(sT\right)}^{2}+1}=\frac{1+\frac{2sT}{\pi }\log\left(sT\right)}{{\left(sT\right)}^{2}+1}.$$

The proposed distribution has a simple structure based on power law and its validity is in the non-Poisson ranges 0<α<2. Using the property $L\left[\exp\left[\pm i\omega_{1}t\right]f\left(t\right)\right]=\hat{f}\left(s\mp i\omega_{1}\right)$, defining the new Laplace variables, $v=s-i\omega_{1}$, we can reduce the inverse Laplace problem of Equation (19) the following inversion Laplace transform

(29) Re1+ψ^v+2iω1Ψ^v1−ψ^v+2iω1ψ^v.

Since we are interested in the asymptotic limit, taking the limit for v→0 we may invert Equation (29). We find for the asymptotic expression

(30) $$\begin{array}{c} \langle \exp\left[i\omega_{1}\int_{0}^{t}\;\;\xi \left(u\right)du\right]\rangle \approx A\frac{\exp\left[i\omega_{1}t+\phi \right]}{t^{\alpha }} \end{array}$$

where *A* and ϕ are constant depending on $\omega_{1},T,\alpha$. In the region 0<α<1 there is no dependence on the parameter *T* and we obtain

(31) expiω1∫0tξ(u)du≈2Reexp(iω1t)Eα−(2iω1t)α≈πJα−12(ω1t)Γ(1−α)(2ω1t)α−12

where $E_{\alpha }\left[-{\left(2i\omega_{1}t\right)}^{\alpha }\right]$ is the Mittag–Leffler function defined as

$$E_{\alpha }\left[z\right]=\sum_{n=0}^{\infty }\frac{z^{n}}{\Gamma (n\alpha +1)},$$

and $J_{\nu }\left(z\right)$ is the Bessel function of the first kind. From an asymptotic point of view, all the expressions contained in Equations (30) and (31) have the same accuracy. The advantage of the expression written as in the last line of Equation (31) is that in the case α=1/2, it provides an exact expression, i.e.

(32) 〈vx(t)〉=v0Imexpiω0texpiω1∫0tξ(u)du=v0sinω0tJ0ω1t,

(33) 〈vy(t)〉=v0Reexpiω0texpiω1∫0tξ(u)du=v0cosω0tJ0ω1t.

This can be directly checked using the distribution P(x,t) derived by Lamperti [31,32], and which describes the distribution associated with Equation (15) for 0<α<1. Integrating Equations (32) and (33) the result with respect to time, we obtain

(34) $$\begin{array}{ccc} & & \langle x\left(t\right)\rangle =v_{0}\int_{0}^{t}\sin\left[\omega_{0}t\right]J_{0}\left[\omega_{1}t\right]dt+x_{0}, \end{array}$$

(35) $$\begin{array}{ccc} & & \langle y\left(t\right)\rangle =v_{0}\int_{0}^{t}\cos\left[\omega_{0}t\right]J_{0}\left[\omega_{1}t\right]dt+y_{0} \end{array}$$

Asymptotically we have

(36) $$\begin{array}{ccc} & & x\left(\infty \right)=\left\{\begin{array}{cc} \frac{v_{0}}{\sqrt{\omega_{0}^{2}-\omega_{1}^{2}}}+x_{0}, & \mathrm{if}\;\omega_{1}<\omega_{0},\\ x_{0}, & \mathrm{if}\;\omega_{0}<\omega_{1}. \end{array}\right. \end{array}$$

(37) $$\begin{array}{ccc} & & y\left(\infty \right)=\left\{\begin{array}{cc} \frac{v_{0}}{\sqrt{\omega_{1}^{2}-\omega_{0}^{2}}}, & \mathrm{if}\;\omega_{0}<\omega_{1},\\ y_{0}, & \mathrm{if}\;\omega_{1}<\omega_{0}. \end{array}\right. \end{array}$$

The resonant case $\omega_{1}=\omega_{0}$ generates diverging average positions

(38) $$\begin{array}{ccc} & & \langle x\left(t\right)\rangle =v_{0}t\left[\sin\left(\omega_{0}t\right)J_{0}\left(\omega_{0}t\right)-\cos\left(\omega_{0}t\right)J_{1}\left(\omega_{0}t\right)\right]+x_{0}, \end{array}$$

(39) $$\begin{array}{ccc} & & \langle y\left(t\right)\rangle =v_{0}t\left[\sin\left(\omega_{0}t\right)J_{1}\left(\omega_{0}t\right)+\cos\left(\omega_{0}t\right)J_{0}\left(\omega_{0}t\right)\right]+y_{0}. \end{array}$$

**Figure 1:**
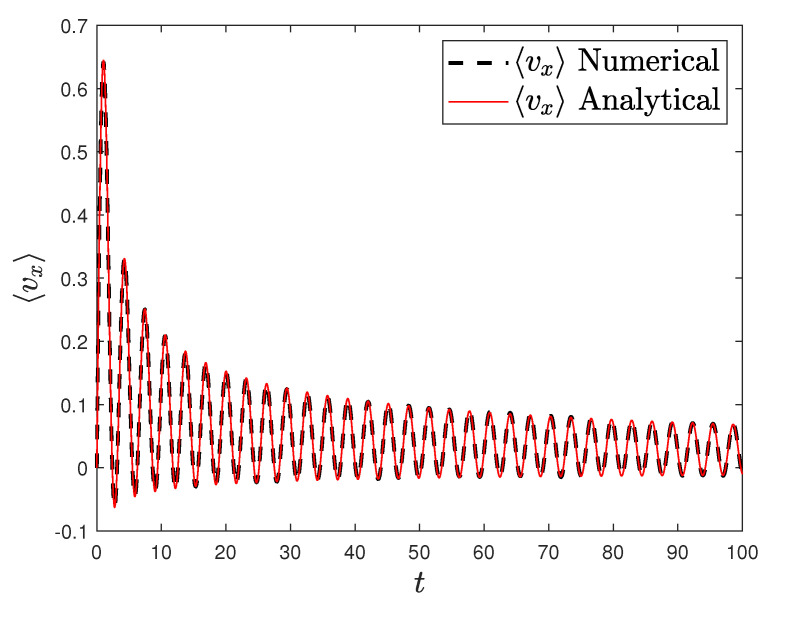
Plot of the analytical (redline) and the numerical solution of the average velocity along the *x* axis with α=0.5, T=0.001, $\omega_{0}=\omega_{1}=1$. Number of realizations 200k.

**Figure 2:**
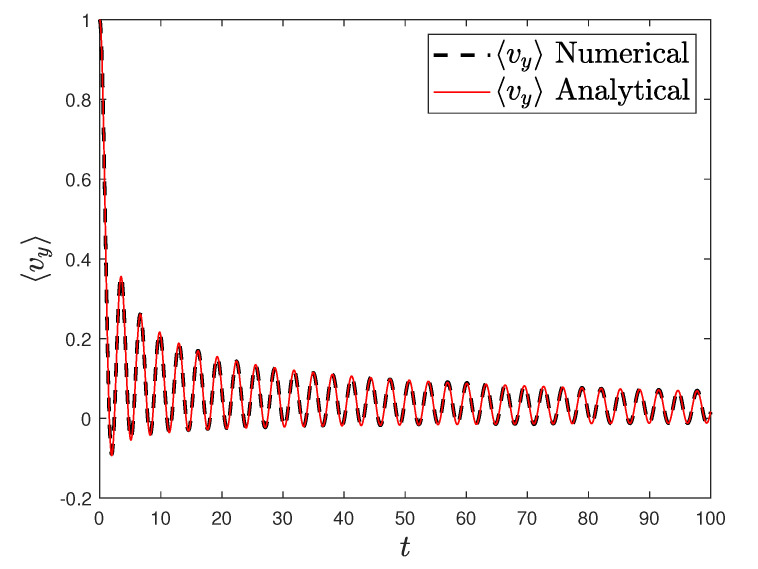
Plot of the analytical (redline) and the numerical solution of the average velocity along the *y* axis with α=0.5, T=0.001, $\omega_{0}=\omega_{1}=1$. Number of realizations 200k.

**Figure 3:**
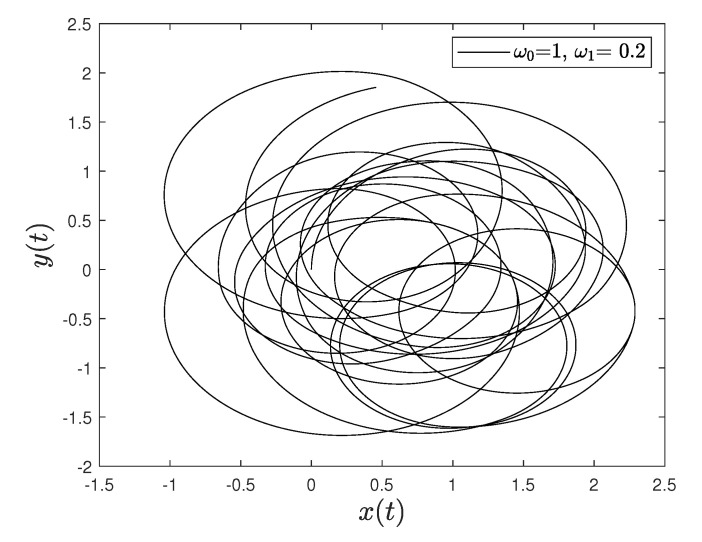
Single realization of the trajectory of a charge in the Poisson case. The values of the parameters are γ=1, $\omega_{0}=1$, $\omega_{1}=0.2$ and t=100.

**Figure 4:**
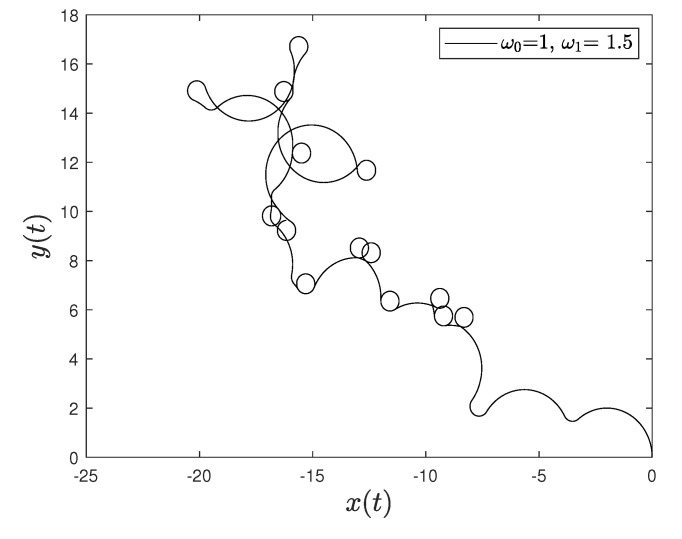
Single realization of the trajectory of a charge in the Poisson case. The values of the parameters are γ=1, $\omega_{0}=1$, $\omega_{1}=1.5$ and t=100.

**Figure 5:**
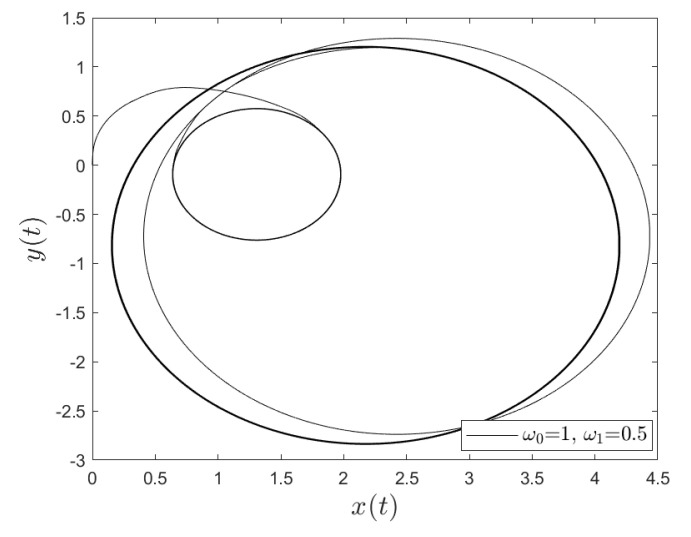
Single realization of the trajectory of a charge in the non-Poisson case. The values of the parameters are α=0.5, T=0.001, $\omega_{0}=1$, $\omega_{1}=0.5$ and t=500.

**Figure 6:**
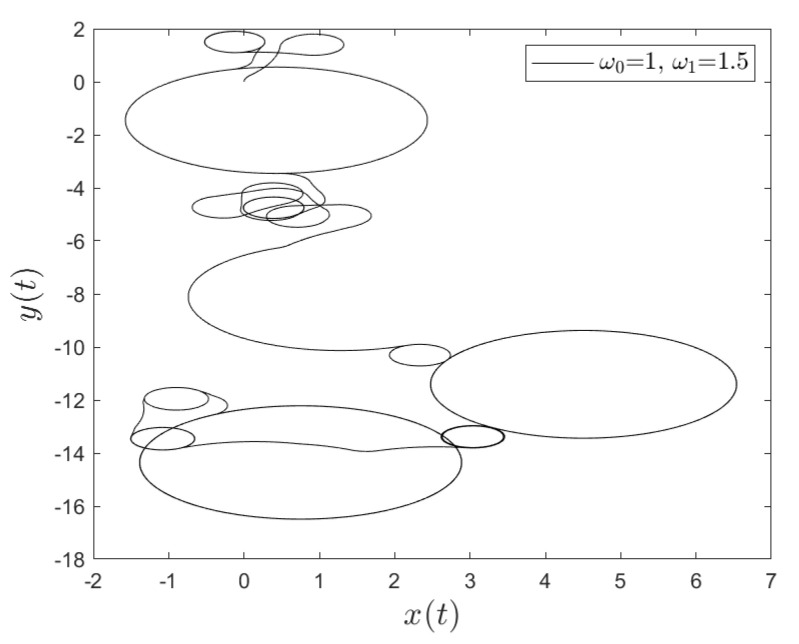
Single realization of the trajectory of a charge in the non-Poisson case. The values of the parameters are α=0.5, T=0.001, $\omega_{0}=1$, $\omega_{1}=1.5$ and t=500.

**Figure 7:**
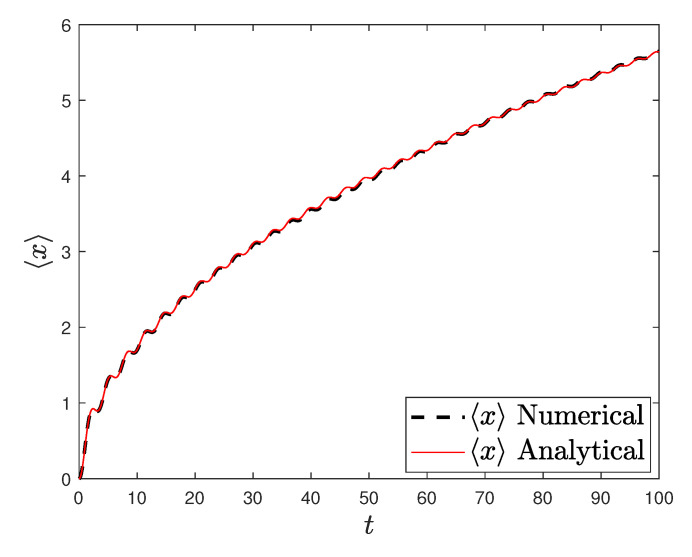
Plot of the analytical (redline) and the numerical solution of the average position 〈x〉 with α=0.5, T=0.001, $\omega_{0}=\omega_{1}=1$, and $x_{0}=0$. Number of realizations 200k.

**Figure 8:**
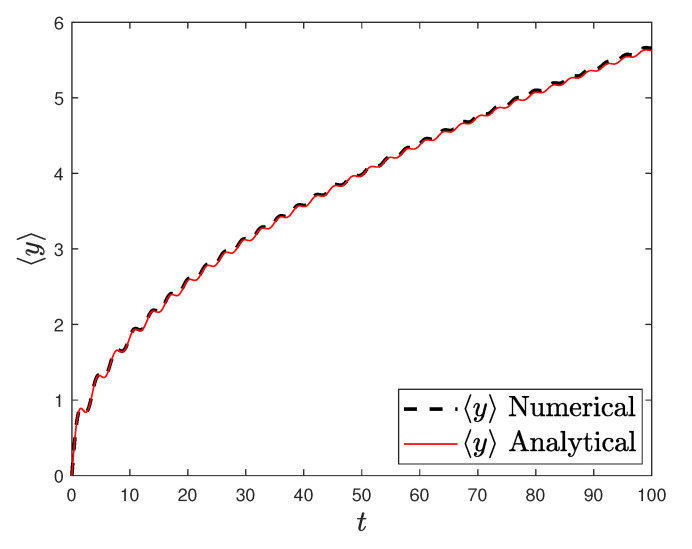
Plot of the analytical (redline) and the numerical solution of the average position 〈y〉 with α=0.5, T=0.001, $\omega_{0}=\omega_{1}=1$, and $y_{0}=0$. Number of realizations 200k.

**Figure 9:**
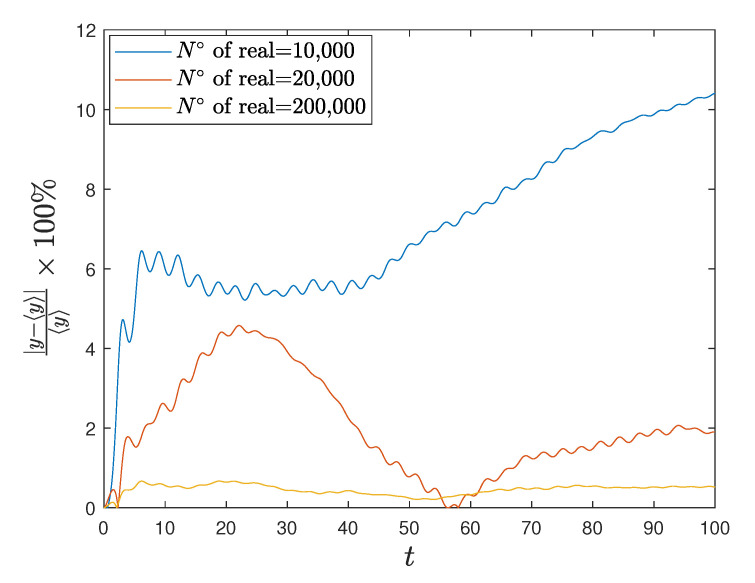
Plot of the percent error. On the *x* axis the time while on the *y* axis the percentage error between numerical and analytical solution. The different curves are obtained using different number of realizations.

Figure 1 and Figure 2 show the comparison between analytical results and numerical simulations for the quantities $\langle v_{x}\left(t\right)\rangle$ and $\langle v_{y}\left(t\right)\rangle$. Figure 3, Figure 4, Figure 5 and Figure 6 show single realizations of the stochastic trajectories, and Figure 7 and Figure 8 show the comparison between analytical results and numerical simulations for the quantities 〈x(t)〉 and 〈y(t)〉. Finally, Figure 9 shows the percent error as a function of the number of the realizations. The error decreases starting from 10% (blu line, 10k realizations) to <1% (yellow line, 200k realizations)

## 4. Diffusion

In this section, we will evaluate $\langle r^{2}\rangle =\langle x^{2}+y^{2}\rangle$ in the Poissonian and non-Poisson case. Using Equations (11) and (12) we have

(40) $$\begin{array}{ccc} & & x^{2}\left(t\right)=\int_{0}^{t}\int_{0}^{t}v_{x}\left(t_{1}\right)v_{x}\left(t_{2}\right)dt_{1}dt_{2}=v_{0}^{2}\int_{0}^{t}\int_{0}^{t}\sin\phi_{t_{1}}\sin\phi_{t_{2}}dt_{1}dt_{2} \end{array}$$

(41) $$\begin{array}{ccc} & & y^{2}\left(t\right)=\int_{0}^{t}\int_{0}^{t}v_{y}\left(t_{1}\right)v_{y}\left(t_{2}\right)dt_{1}dt_{2}=v_{0}^{2}\int_{0}^{t}\int_{0}^{t}\cos\phi_{t_{1}}\cos\phi_{t_{2}}dt_{1}dt_{2} \end{array}$$

where we set

$$\phi_{t}=\int_{0}^{t}\omega \left(u\right)du=\omega_{0}t+\omega_{1}\int_{0}^{t}\xi \left(u\right)du.$$

Consequently

(42) $$x^{2}+y^{2}=r^{2}=v_{0}^{2}\int_{0}^{t}\int_{0}^{t}\cos\left(\phi_{t_{1}}-\phi_{t_{2}}\right)dt_{1}dt_{2}.$$

Our goal is to evaluate the quantity $\langle r^{2}\rangle$. For the sake of compactness let us define the complex quantity

(43) $$r_{2}\left(t\right)=v_{0}^{2}\int_{0}^{t}\int_{0}^{t}\exp\left[i(\phi_{t_{1}}-\phi_{t_{2}})\right]dt_{1}dt_{2},$$

and take its time derivative

(44) $$\begin{array}{c} \frac{\partial }{\partial t}r_{2}\left(t\right)=2v_{0}^{2}\int_{0}^{t}\exp\left[i\omega_{0}\left(t-t_{1}\right)\right]\langle \exp\left[i\omega_{1}\int_{t_{1}}^{t}\xi \left(u\right)du\right]\rangle dt_{1}. \end{array}$$

For a Poisson process, with exponential waiting times distribution and correlation, the distribution of the first observed jump/event is the same as that of any other following event [24], this means that when averaging over the fluctuations, shifting the time origin, will not affect the result. For a generic non-Poissonian process, but with a finite time scale, this remains a good approximation in the long-time limit, so that we may re-write Equation (44) as

(45) $$\begin{array}{c} \frac{\partial }{\partial t}r_{2}\left(t\right)\approx 2v_{0}^{2}\int_{0}^{t}\exp\left[i\omega_{0}\left(t-t_{1}\right)\right]\langle \exp\left[i\omega_{1}\int_{0}^{t-t_{1}}\xi \left(u\right)du\right]\rangle dt_{1}. \end{array}$$

Formally, for t→∞, Equation (45) is the Laplace transform of the averaged function where $s\to i\omega_{0}$. Using the result of Equation (19), we may write

(46) $$\begin{array}{ccc} & & \frac{\partial }{\partial t}r_{2}\left(t\right)\approx v_{0}^{2}\left[\frac{\left(1+\hat{\psi }\left(i\omega_{0}-i\omega_{1}\right)\right)\hat{\Psi }\left(i\omega_{0}+i\omega_{1}\right)}{1-\hat{\psi }\left(i\omega_{0}-i\omega_{1}\right)\hat{\psi }\left(i\omega_{0}+i\omega_{1}\right)}+\right.\\ & & \left.\frac{\left(1+\hat{\psi }\left(i\omega_{0}+i\omega_{1}\right)\right)\hat{\Psi }\left(i\omega_{0}-i\omega_{1}\right)}{1-\hat{\psi }\left(i\omega_{0}-i\omega_{1}\right)\hat{\psi }\left(i\omega_{0}+i\omega_{1}\right)}\right], \end{array}$$

namely a constant. We deduce that for the Poisson and the non-Poisson case, but with 1<α<2, we have ordinary diffusion, i.e.,

(47) 〈r2〉=Re[r2(t)]∝t

Please note that in the Poisson case the approximation (45) is actually an exact expression and $\langle \exp\left[i\omega_{1}\int_{0}^{t-t_{1}}\xi \left(u\right)du\right]\rangle$ is given by Equation (21). The integration of (45) and the subsequent integration to obtain $\langle r^{2}\rangle$ does not present difficulties being the integral functions exponential functions. Neglecting the transient due to the exponentials with negative real part, the asymptotic expression for $\langle r^{2}\rangle$ reads as

(48) $$\langle r^{2}\rangle \approx \frac{2\gamma v_{0}^{2}\omega_{1}^{2}}{\gamma^{2}\omega_{0}^{2}+{\left(\omega_{0}^{2}-\omega_{1}^{2}\right)}^{2}}t,\;\;t\to \infty .$$

**Figure 10:**
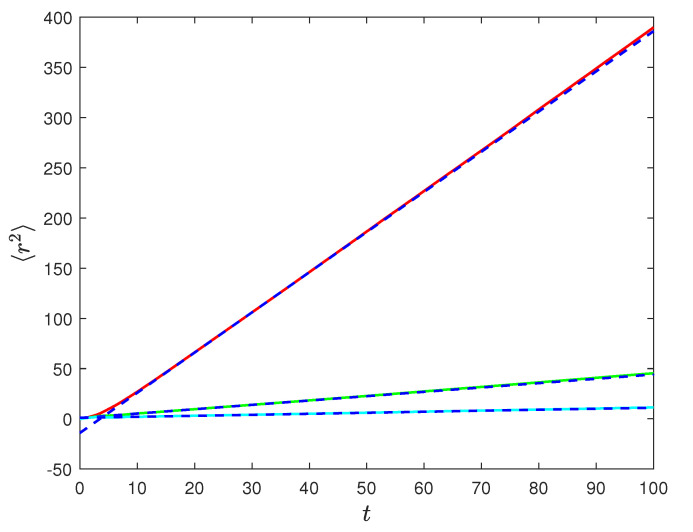
Plots of $\langle r^{2}\rangle$ in the Poisson case as derived from Equation (48) compared with numerical simulation. Red continuous line and blue dashed line refer to the analytical and numerical derivation respectively for parameter values γ=0.5, $\omega_{0}=\omega_{1}=1$. Green continuous line and blue dashed line are the analytical and numerical derivation for parameters γ=0.5, $\omega_{0}=1,\;\;\omega_{1}=2$. Cyan continuous line and blue dashed line are analytical and numerical derivation for γ=0.5, $\omega_{0}=2,\;\;\omega_{1}=1$. Number of realizations 200k.

**Figure 11:**
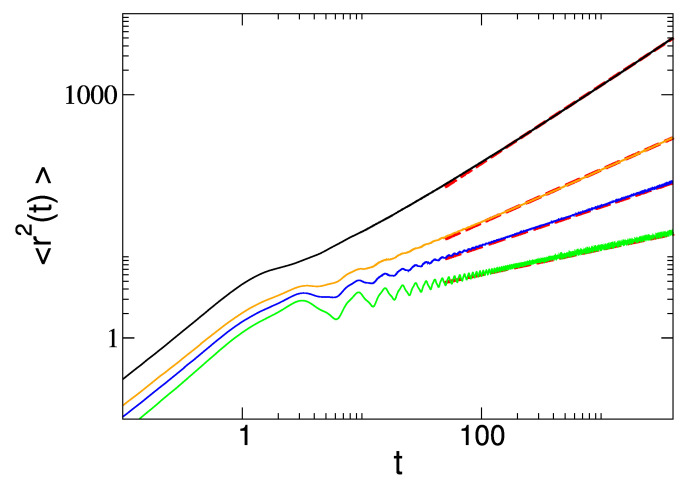
Plots of mean-squared displacement $\langle r^{2}\left(t\right)\rangle$ as a function of time. Black, orange, blue and green line correspond respectively to α=1.5,0.7,0.5,0.3. We have chosen $\omega_{0}=2$ and $\omega_{1}=1$ and for the waiting-time distribution $\psi \left(t\right)\propto {\left(t/T\right)}^{-\alpha -1}$ with T=0.001. Superimposed red dashed lines represent guide for the eye for the asymptotic behavior as derived in the text, i.e., $\langle r^{2}\left(t\right)\rangle \propto t$ for 1<α<2 ( black line case) and $\langle r^{2}\left(t\right)\rangle \propto t^{\alpha }$ for 0<α<1 (all other plots).

The diffusion is faster at resonance, when $\omega_{0}=\omega_{1}$. The numerical check for the quantity $\langle r^{2}\rangle$, obtained integrating the uniform circular motion between switches of the magnetic field value, is shown in Figure 10 (Poisson case) and Figure 11 (1<α<2). The agreement with the analytical result, Equation (47), is remarkable.

When 0<α<1 there is not a finite time scale, and we cannot use the approximation given by Equation (45). It is important to notice that while in the Poissonian case the distribution of the first observed event/jump coincides with the distribution ψ(t) of any other event, in the non-Poissonian case it will be different from ψ(t) and it will be function also of the time $t_{a}<0$, the time at which the system is prepared, in other words, the distribution of the first observed event will have a two-times dependence $\psi (t,t_{a})$. The distribution of the first jump must be considered, and we are forced to use the full formula of Ref. [24], i.e.,

(49) $$\begin{array}{ccc} & & L\left[\int_{0}^{t}\exp\left[i\omega_{0}\left(t-t_{1}\right)\right]\langle \exp\left[i\omega_{1}\int_{t_{1}}^{t}\xi \left(u\right)du\right]\rangle dt_{1}\right]=\\ & & \frac{1}{2}\frac{\left[{\hat{f}}_{-}\left(s\right)+{\hat{f}}_{+}\left(s\right)\hat{\psi }\left(s-i\delta_{-}\right)\right]\hat{\Psi }\left(s-i\delta_{+}\right)}{1-\hat{\psi }\left(s-i\delta_{+}\right)\hat{\psi }\left(s-i\delta_{-}\right)}+\frac{1}{2}{\hat{F}}_{+}\left(s\right)+\\ & & \frac{1}{2}\frac{\left[{\hat{f}}_{+}\left(s\right)+{\hat{f}}_{-}\left(s\right)\hat{\psi }\left(s-i\delta_{+}\right)\right]\hat{\Psi }\left(s-i\delta_{-}\right)}{1-\hat{\psi }\left(s-i\delta_{+}\right)\hat{\psi }\left(s-i\delta_{-}\right)}+\frac{1}{2}{\hat{F}}_{-}\left(s\right) \end{array}$$

where $\delta_{\pm }=\omega_{0}\pm \omega_{1}$, and

(50) $${\hat{f}}_{\pm }\left(s\right)=L\left[\int_{0}^{t}f\left(t-t_{1},t_{1}\right)\exp\left[i\delta_{\pm }\left(t-t_{1}\right)\right]dt_{1}\right]=\frac{\hat{\psi }\left(s\right)-\hat{\psi }\left(s-i\delta_{\pm }\right)}{-i\delta_{\pm }\left[1-\hat{\psi }\left(s-i\delta_{+}\right)\right]}$$

being $f(\tau ,t_{1})$ the conditional probability density that, fixed at time $t_{1}$, the first next switching event of the variable ξ(t) occurs at time $t_{1}+\tau$. It is important to notice that differently from the Poisson case, this distribution is different from the distribution ψ(t) of any other event. It coincides with ψ only for the Poisson case. Analogously

(51) $${\hat{F}}_{\pm }\left(s\right)=L\left[\int_{0}^{t}F\left(t-t_{1},t_{1}\right)\exp\left[i\delta_{\pm }\left(t-t_{1}\right)\right]dt_{1}\right]=\frac{1/s-{\hat{f}}_{\pm }\left(s\right)}{s-i\delta_{\pm }}$$

where

$$F\left(\tau ,t_{1}\right)=1-\int_{0}^{\tau }f\left(\tau^{\prime },t_{1}\right)d\tau^{\prime }$$

is the conditional probability that, fixed $t_{1}$, no switch occurs between $t=t_{1}$ and $t=t_{1}+\tau$. Using distribution (27) or in alternative the Mittag–Leffler distribution, we may write for the inverse Laplace transform of ${\hat{f}}_{\pm }\left(s\right)$ the following

(52) $$\begin{array}{ccc} & & f_{\pm }\left(t\right)=L^{-1}\left[{\hat{f}}_{\pm }\left(s\right)\right]=-\frac{1}{\delta_{\pm }}\frac{d}{dt}E_{\alpha }\left[-{\left(\frac{t}{T}\right)}^{\alpha }\right]-\frac{1}{\delta_{\pm }T}\frac{1}{\Gamma (\alpha )}{\left(\frac{t}{T}\right)}^{\alpha -1}-\\ & & \frac{1}{\delta_{\pm }T\Gamma \left(\alpha \right)}\int_{0}^{t}\exp\left[i\delta_{\pm }\left(t-t_{1}\right)\right]{\left(\frac{t-t_{1}}{T}\right)}^{\alpha -1}\frac{d}{dt_{1}}E_{\alpha }\left[-{\left(\frac{t_{1}}{T}\right)}^{\alpha }\right]dt_{1} \end{array}$$

From Equation (52), one can also understand why the contribution of the first jump/ event becomes crucial for a non-Poissonian process for 0<α<1. In this regime, differently from the Poisson regime, not only is such distribution different from that of any following event [24], but it also becomes dominant asymptotically. Analyzing Equation (51) from an asymptotic point of view, namely sT→0 and δT≪1 we deduce that contribution to the diffusion is given by $F_{\pm }$.

(53) $$\begin{array}{c} F_{\pm }\left(t\right)=\frac{i}{\delta_{\pm }}-\frac{i\exp[i\delta_{\pm }t]}{\delta_{\pm }}+\int_{0}^{t}\exp\left[i\delta_{\pm }\left(t-t_{1}\right)\right]f\left(t_{1}\right)dt_{1} \end{array}$$

As $f_{\pm }\left(t\right)$ is a function decaying with time we may approximate $F_{\pm }\left(t\right)$ for t→∞ (see Ref. [27] for more details)

(54) $$\begin{array}{c} F_{\pm }\left(t\right)\approx \frac{i}{\delta_{\pm }}-\frac{i\exp[i\delta_{\pm }t]}{\delta_{\pm }}+\exp\left[i\delta_{\pm }t\right]{\hat{f}}_{\pm }\left(i\delta_{\pm }\right). \end{array}$$

The contribution of $F_{\pm }\left(t\right)$ to $\langle r^{2}\left(t\right)\rangle$ generates constant and oscillating terms while the dominant term is a power law. Indeed, we have

(55) $$\begin{array}{c} \langle r^{2}\left(t\right)\rangle ∼2v_{0}^{2}Re\left[A\right]\frac{1}{\Gamma [\alpha ]\Gamma [\alpha -1]}{\left(\frac{t}{T}\right)}^{\alpha },\;\;\mathrm{for}\;\;t\to \infty . \end{array}$$

The analytical result is obtained expanding for small *s* the Laplace transform of the expression for the derivative of $\langle r^{2}\left(t\right)\rangle$, neglecting the terms ${\hat{F}}_{\pm }\left(s\right)$ for the reason stated above. The following expression gives the coefficient *A*

(56) $$A=\frac{1}{1-{\hat{\psi }}_{+}{\hat{\psi }}_{-}}\left[\frac{{\hat{\Psi }}_{+}\left(1-{\hat{\psi }}_{+}\right){\hat{\psi }}_{-}}{i\delta_{+}}+\frac{{\hat{\Psi }}_{+}\left(1-{\hat{\psi }}_{-}\right)}{i\delta_{-}}\right]-\frac{1-{\hat{\psi }}_{+}}{\delta_{+}^{2}}+\left(+↔-\right)$$

with ${\hat{\Psi }}_{\pm }$ and ${\hat{\psi }}_{\pm }$ the Laplace transform of the respective functions Ψ(t) and ψ(t) evaluated in $s=-i\delta_{\pm }$ and the last term within round brackets is obtained from previous terms by exchanging + and − subscripts. The numerical check is shown in Figure 11.

## 5. Results

To summarize, we have introduced a complete framework to describe the motion of a charged particle in a fluctuating magnetic field. We considered both ergodic and non-ergodic fluctuations of the magnetic field. We find that in the case of ergodic fluctuations the diffusion is asymptotically normal, while for non-ergodic fluctuations, we find anomalous diffusion properties. The diffusion is characterized by the mean-squared displacement, which we derived analytically and confirmed numerically for both regimes as illustrated in Figure 10 and Figure 11. In the case of non-Poisson fluctuations, we provide analytical formulae in Laplace transform from which we extract the asymptotic time behavior for the mean-squared displacement.

## 6. Discussion and Conclusions

The problem of the motion of a charged particle in fluctuating magnetic field has not been investigated when fluctuations of the field have non-ordinary statistical properties despite its physical interest and possible applications e.g., to plasma models. This paper starts filling this gap by considering dichotomic fluctuations with both Poisson and non-Poisson properties. For the Poisson case, we find the driven equation for the probability density. On the contrary, the equation for probability density function in the non-ordinary statistics case is still an open problem. We developed a theoretical framework for the first and second moment of the particle position and afforded both an analytical and numerical description. We neglected in this framework the effect of induced electric field due to the variations of the magnetic field, which we leave out for further investigation in an upcoming publication. Interestingly, we find that diffusion is normal either when fluctuations are Poisson or non-Poisson but with a finite mean time, differently from the standard case of continuous-time random walk (CTRW) which shows anomalous diffusion behavior for power-law distribution with finite mean time (either in the velocity or jump model) [33]. When the fluctuations time scale diverges, i.e., for non-ergodic fluctuations, an anomalous diffusion regime emerges, again differently from standard CTRW where a ballistic regime applies for power-law distributions with diverging mean time. This difference is related to the fact that the length of the CTRW jumps is proportional to the elapsed time while in the case studied in the paper, the particle is forced to move along a circular trajectory. Thus, the jumps cannot exceed the radius of the circumference, causing the relevant differences between the two diffusion processes.

## Data Availability

Data sharing not applicable.
